# The ASPECT Hydrocephalus System: a non-hierarchical descriptive system for clinical use

**DOI:** 10.1007/s00701-022-05412-6

**Published:** 2022-11-24

**Authors:** Joachim Birch Milan, Thorbjørn Søren Rønn Jensen, Nicolas Nørager, Sarah Skovlunde Hornshøj Pedersen, Casper Schwartz Riedel, Nikolaj Malthe Toft, Ahmed Ammar, Mansoor Foroughi, André Grotenhuis, Andrea Perera, Harold Rekate, Marianne Juhler

**Affiliations:** 1 Copenhagen CSF Study Group, Copenhagen, Denmark; 2 Department of Neurosurgery 6031, Rigshospitalet, Inge Lehmanns Vej 6, Copenhagen, DK 2100 Denmark; 3 Department of Neurosurgery, King Fahd University Hospital, Imam Abdulrahman Bin Faisal University, Dammam, Saudi Arabia; 4 European Association of Neurosurgical Societies (EANS) CSF Task Force, Brussels, Belgium; 5 Department of Neurosurgery, Wellington Hospital, London, UK; 6 Department of Neurosurgery, Radboud University Nijmegen Medical Centre, Nijmegen, Holland Netherlands; 7 Department of Basic and Clinical Neuroscience, Kings College London, Maurice Wohl Clinical Neuroscience Institute, London, UK; 8 Department of Neurosurgery, Hofstra Northwell School of Medicine in Hempstead, Hempstead, NY USA; 9 Department of Clinical Medicine, University of Copenhagen, Copenhagen, Denmark

**Keywords:** Hydrocephalus, ASPECT Hydrocephalus System

## Abstract

In patients with hydrocephalus, prognosis and intervention are based on multiple factors. This includes, but is not limited to, time of onset, patient age, treatment history, and obstruction of cerebrospinal fluid flow. Consequently, several distinct hydrocephalus classification systems exist. The International Classification of Diseases (ICD) is universally applied, but in ICD-10 and the upcoming ICD-11, hydrocephalus diagnoses incorporate only a few factors, and the hydrocephalus diagnoses of the ICD systems are based on different clinical measures. As a consequence, multiple diagnoses can be applied to individual cases. Therefore, similar patients may be described with different diagnoses, while clinically different patients may be diagnosed identically. This causes unnecessary dispersion in hydrocephalus diagnostics, rendering the ICD classification of little use for research and clinical decision-making. This paper critically reviews the ICD systems for scientific and functional limitations in the classification of hydrocephalus and presents a new descriptive system. We propose describing hydrocephalus by a system consisting of six clinical key factors of hydrocephalus: A (anatomy); S (symptomatology); P (previous interventions); E (etiology); C (complications); T (time–onset and current age). The “*ASPECT Hydrocephalus System*” is a systematic, nuanced, and applicable description of patients with hydrocephalus, with a potential to resolve the major issues of previous classifications, thus providing new opportunities for standardized treatment and research.

**Supplementary Information:**

The online version contains supplementary material available at 10.1007/s00701-022-05412-6.

## Introduction

Several factors define prognosis and the course of treatment for patients with hydrocephalus. Age, time of onset, etiology, previous treatment, and obstruction of cerebrospinal fluid (CSF) flow are all influential factors [21, 25].

Hydrocephalus has traditionally been classified as obstructive or communicating, based on Dandy and Blackfan’s experiments in the 1910s [4]. Dandy made the distinction based on “the presence or the absence of communication between the ventricles and the subarachnoid space” [5].

Dandy’s work is recognized as pioneering and is still the way many neurosurgeons think when choosing between endoscopic third ventriculostomy (ETV) or ventricular shunting [16, 17, 30, 39]. However, the simplicity of the dichotomous classification has been challenged [9, 27], and several proposals for additional or alternative classification factors have been published [1, 7, 8, 21, 23, 24, 26, 29, 37]. The overall purpose of these classifications is clinical usefulness and/or research. However, since they are constructed on just one or two clinical factors, and hydrocephalus is a multifactor disease, the exclusive use of these classifications carries a risk of disregarding other clinically meaningful factors. Still, some of these additional classifications are incorporated in contemporary International Classification of Diseases (ICD)-systems [11].

**Fig. 1:**
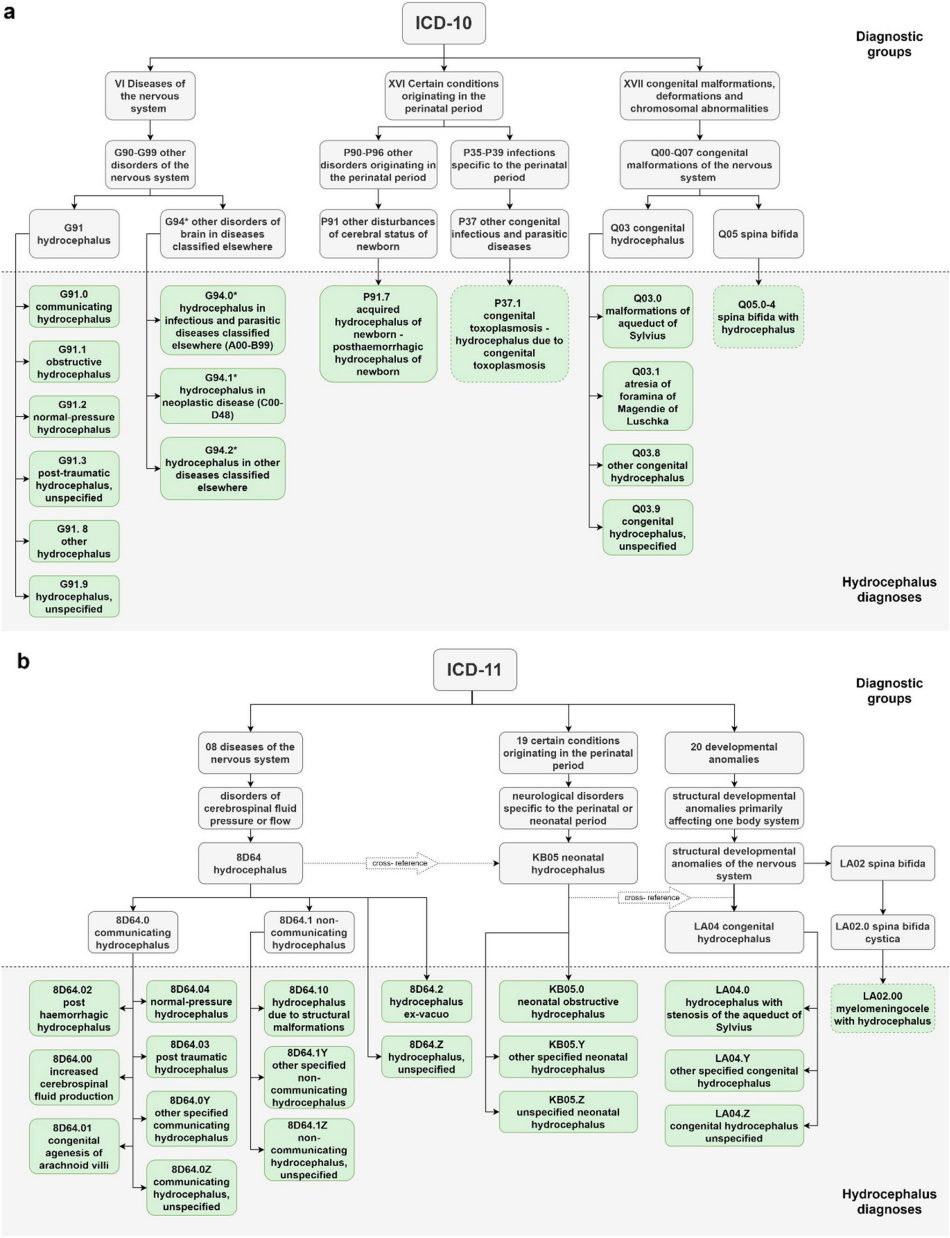
ICD-10 is the current version (in the US-modified ICD-10-CM). ICD-11 released in 2018 is planned to replace ICD-10 in 2022 [34]. Both ICD-10 and ICD-11 have logical and functional limitations. Consequently, none of the diagnoses may be appropriate for some patients, while multiple diagnoses might apply to other patients. ICD-10: in the G9-groups, there is a forced choice between anatomical communication (G91.0 and G91.1), underlying etiology (G91.3, G94.0, G94.1, G94.2), or NPH—a syndrome defined by a mixture of clinical symptomatology (G91.2) although considerations of anatomy, symptomatology and etiology will apply to any patient. When classifying congenital and neonatally acquired hydrocephalus, the classifier must choose between two specific underlying causes (P37.1 and P91.7), three specified congenital developmental/anatomical abnormalities (Q03.0, Q03.1, and Q06), or other (Q03.8). ICD-11 dichotomizes into communicating vs. non-communicating hydrocephalus as the primary classification step in non-neonate patients. The second-tier classification includes a mixture of underlying etiology, structural anomalies, and clinical symptomatology (NPH) on the same hierarchical level. The system flaws are thus a forced association between anatomical presentations and certain etiologies and a forced choice between clinical factors which may all apply to any patient. Both systems include very rare diagnoses as additional options (congenital toxoplasmosis P37.1; hydrocephalus caused by increased CSF production” 8D64.00) while excluding other more common entities, e.g., bacterial infections

Today, Dandy’s classification is still a significant constituent of the globally used ICD [11]. However, the ICD classification system initially built for epidemiology registration is not useful for guiding clinical decisions [6]. For many years, it has been used for managing the health care economy, but because of logical flaws, it carries a risk for uncertain classification or misclassification even for these purposes (Fig. 1).

In this paper, we propose a multifactor approach to describe hydrocephalus and thus accommodate the complexity of the disease. We have constructed a descriptive system of parallel factors without a forced hierarchy to recognize that factors may be equally important or of different importance in different patients. The primary aim is that this system will be useful for clinical management, and a secondary aim is that the system will be useful for research purposes.

### The ASPECT Hydrocephalus System

The system proposed in this paper is based on the following original definition of hydrocephalus: Hydrocephalus is a pathological state in which abnormal cerebrospinal fluid dynamics causes enlargement of one or more of the CSF compartments of the brain. This is in contrast to other definitions ranging from very broad (including cerebral edema [28]) to highly specific (based on a particular theory regarding hydrocephalus pathogenesis—the bulk flow model [29]), and definitions including disorders not directly related to pathological CSF dynamics, e.g., brain edema and hydrocephalus ex vacuo. Importantly, it is insensitive to theories or controversies about the pathogenesis of hydrocephalus.

The proposed system is constructed to fulfill four criteria for the utility of a clinical classification system:

1. *Coverage*—an appropriate diagnosis for every disorder
2. *Reproducibility*—no more than one appropriate diagnosis per disorder
3. *Applicability*—applicable with widely available diagnostic tools without requirement of advanced technology or advanced level of expertise
4. *Informativeness*—insight into the underlying disorder for the patient and the clinician

## Methods

Neurosurgeons with recognized expertise in hydrocephalus were consulted to determine the patient characteristics most relevant to prognosis, clinical decision-making, and future research in hydrocephalus (supplementary). All identified six factors as being important. These six factors were then consolidated in the ASPECT Hydrocephalus System (Table 1). They appear in a non-hierarchal order as there was no consensus on ranking. A set of numbered, predefined answers were added to each factor, providing the opportunity of applying a standardized numerical code. Coding with similar principles has proven useful in other diseases, e.g., TNM (tumor, node, metastasis) classification of malignant tumors [22].

**Table 1:**
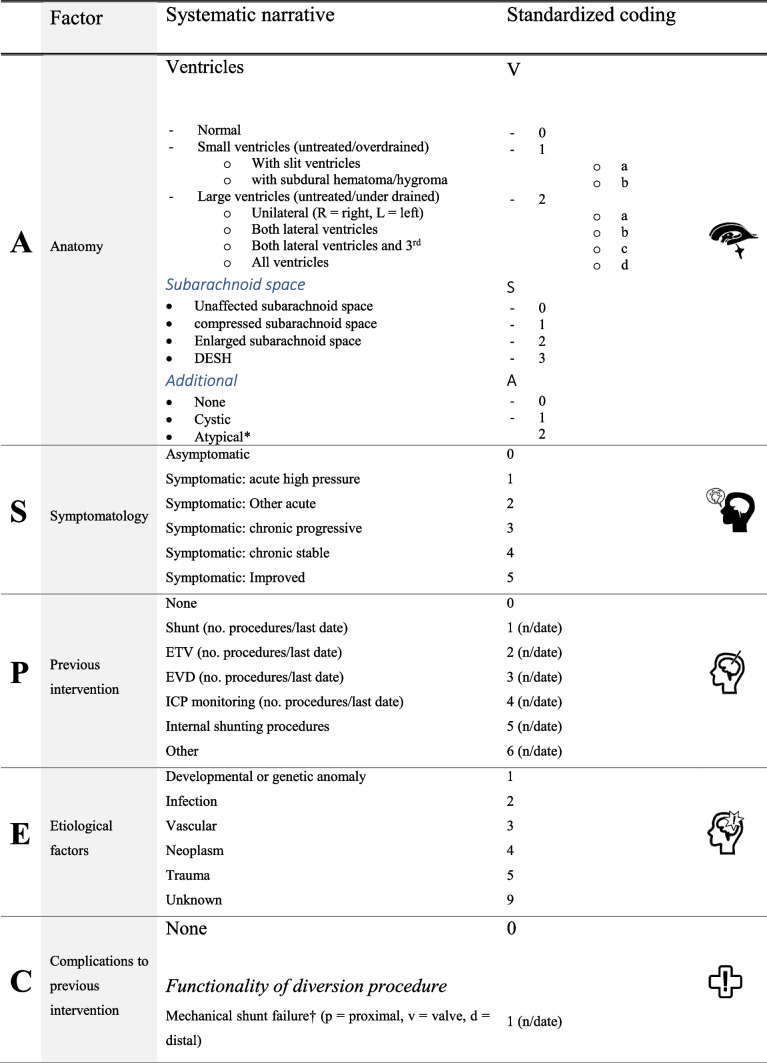

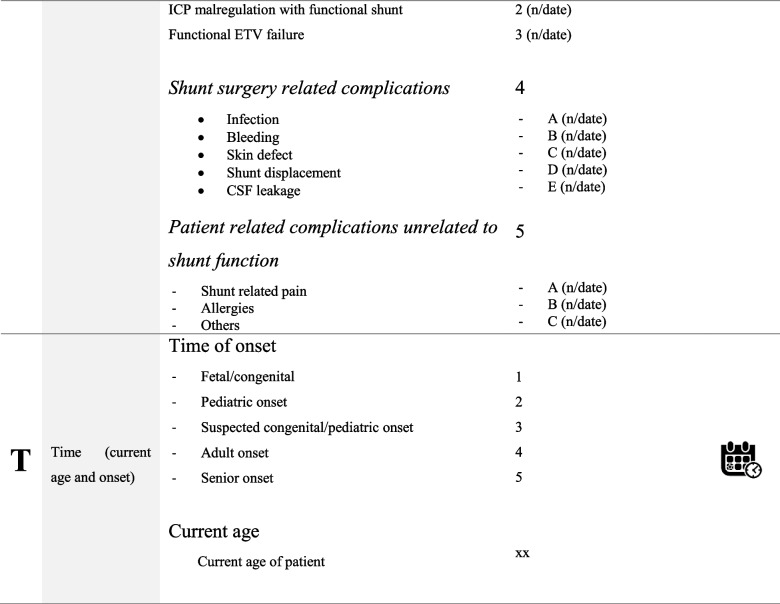
The ASPECT hydrocephalus system the format of factors CT and T look strange here - however, the format seems correct in the PDF version

The intended format is combining the acronym letters with the numeric options; e.g. P1; AV2c/S0/A0. Optional answer for all factors: “9” if the answer is unknown. Alternatively, the factors may be described in prose

^*^Atypical ventriculomegaly, e.g., multiloculated hydrocephalus or hydrocephalus combined with other pathologies

^†^Obstruction, disconnection, tube defect, and valve dysfunction

Development of the ASPECT Hydrocephalus System was an iterative process with several adjustments guided by coding a randomly selected test cohort of 50 patients with hydrocephalus treated at Rigshospitalet (Copenhagen, Denmark) (supplementary). We arrived at the presented system when all 50 patients in the test cohort could be coded by one and only one combination.

### Factor “A”—anatomy

**Fig. 2:**
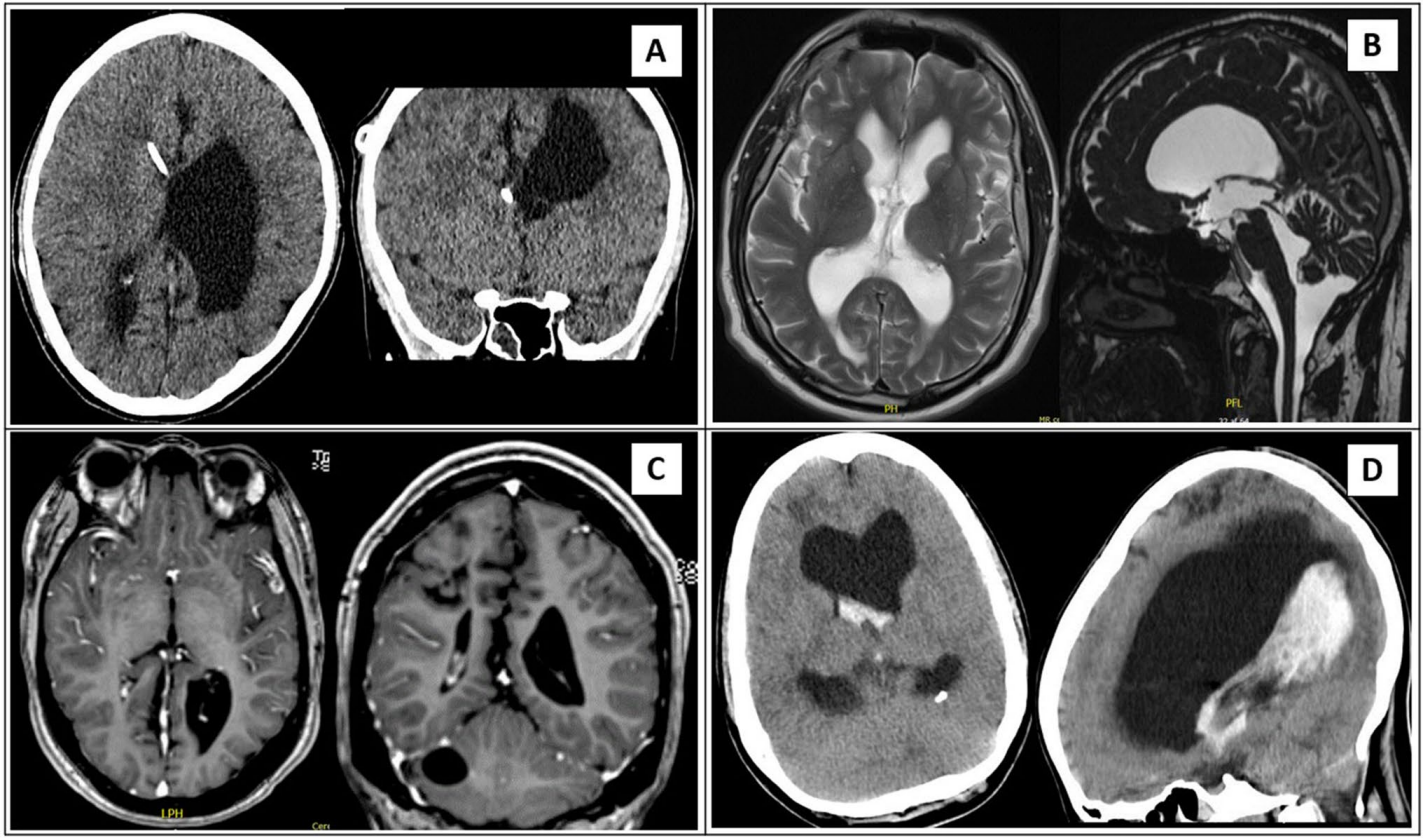
This Figure illustrates four cases of anatomical (**A**) classification of the ASPECT hydrocephalus system. **A** 5-year-old girl with shunt-treated congenital hydrocephalus and asymmetrical ventricular system containing both left-sided ventricular enlargement and right-sided overdrainage. A-classification: V1a/V2a-S0-A0. **B** 54-year-old man with obstructive hydrocephalus with enlarged cisterna magna. He was treated with endoscopic third ventriculostomy. A-classification: V2c-S0-A2. **C** 34-year-old male with myelomeningocele and shunt-treated congenital hydrocephalus with the ventricular drain in the right lateral ventricle. Additionally, the patient has an arachnoid cyst in the right cerebellar hemisphere. A-classification: V0/V2a-S0-A1. **D** 65-year-old male with intraventricular hemorrhage and enlargement of both lateral ventricles with a possible cyst in the right occipital horn. He was treated with EVD. A-classification: V2b-S1-A2

Iterative coding of the test cohort made it clear that description of ventricular anatomy needed to allow diversity in size of ventricular compartments in order to include cases with both universal and local changes in ventricular size. Changes in ventricular size also needed to include symmetrical and asymmetrical variations, e.g., allowing the coding of overdrainage in the shunted lateral ventricle and relative distension of the opposite lateral ventricle. Additionally, a description of extra-ventricular CSF pathways was needed, as some forms of hydrocephalus include expansion or compression of the subarachnoid space. A few patients had more complicated anatomy with single or multiple cysts or other additional pathology relating to the hydrocephalus, which led to the coding amendment for “additional anatomy.” This resulted in a composite coding of “*V*” for ventricular size (0 = normal; 1 = small ventricles (untreated/overdrained); 2 = large ventricles (untreated/under drained) with additional factors a–d for, e.g., symmetry/asymmetry); “*S*” for subarachnoid space (0 = normal; 1 = compressed; 2 = enlarged; 3 = disproportionately enlarged subarachnoid space hydrocephalus (DESH)); and “*A*” for additional anatomical features”. For patients with asymmetric ventricles, right side is presented with R and the left side with L. For details see Table 1 and examples of coding see Fig. 2. Below some illustrative examples from the test cohort are shown:

1. V2d/S0/A0 is the code for a universally dilated ventricular system with an unaffected subarachnoid space and no additional pathology
2. V2c/S1/A0 is the code for symmetrical dilatation of the supratentorial ventricular system with compressed subarachnoid space; typically an aquaductal stenosis
3. V1R/V2L/S0/A0 is the code for an asymmetrical ventricular system with unaffected subarachnoid space and no additional pathology, e.g., unilateral overshunting with compromise of the opposite foramen Monroi

The intention is to encourage a systematic analysis of imaging aiding clinical conclusion and management strategy. It is also intended that this does not require advanced neuro-imaging but can be performed by non-experts and in non-expert institutions. In the above examples, case 1 does not present a definable point of CSF flow restriction and a shunt implant would be treatment of choice; case 2 has signs of increased intracranial pressure (ICP) and a definable point of obstruction which can be by-passed by ETV; case 3 has un-balanced anatomy due to overshunting and could be managed either by changing valve characteristics or by converting treatment to ETV/septostomy if technically possible. It may also be possible to compare coding of previous imaging to current imaging providing a quickly accessible history of the patient’s anatomy.

### Factor “S”—symptomatology

There are several ways to approach a system to describe symptoms. One is grouping symptom constellations according to clinical picture/syndromes (e.g., normal pressure hydrocephalus (NPH)); another is to provide a symptom list; a third is distinguishing between severe and less severe symptoms. We were faced with the challenge that all often apply to clinical situations. However, the ASPECT Hydrocephalus System is not meant to substitute detailed information in the clinical record, and therefore a symptom list was discarded as an option. We defined the intention of this factor as not to provide a diagnosis but rather to arrive at symptom categories aiding the conclusion whether intervention was needed or not—and if needed, treatment was an emergency. It was therefore necessary on one hand to distinguish between high pressure/acute symptoms vs. chronic symptoms and on the other to be able to conclude whether the situation was changed from previously. This resulted in the categories asymptomatic (0), symptomatic: acute high pressure (1), symptomatic: other acute (2), symptomatic: chronic progressive (3), symptomatic: chronic stable (4), symptomatic: improved (5).

### Factor “P”—previous interventions

The treatment options for hydrocephalus are essentially limited to either shunt implantation or endoscopic fenestration. Temporary interventions as externalized drainage (EVD) or ICP monitoring may also be part of the history. The list of intervention types is short and simple: none (0), shunt (1), ETV (2), EVD (3), ICP monitoring (4), internal shunting procedures (5), other (6).

However, as hydrocephalus is a chronic condition, implanted shunts have limited durability, and ETV has a variable success rate, a patient’s history can accumulate one or more intervention types over time. We therefore saw that applying the code “1” for shunt treatment in many cases did not sufficiently contain the intervention history, however, adding a numerator would give a more comprehensive description.

1. P1(1) is the patient with just one shunt implant
2. P1(4)P2(1) is the patient with four shunt surgeries and one ETV
3. P1(10)P 2(1),P3(3),P4(5) is the patient with 10 shunt surgeries, one ETV, three EVDs, and four ICP monitoring surgeries.

It is probably feasible to add the current treatment, e.g., date, valve type/setting and type of the latest shunt surgery.

### Factor “E”—etiology

The chapter division in ICD categorizes diseases into accepted and clinically useful categories, so we decided to adopt this to describe the distribution of underlying causes for hydrocephalus. In our test cohort, we did not encounter problems or doubt by using the following options: developmental or genetic anomaly (1), infection (2), vascular (3), neoplasm (4), trauma (5), unknown (9). We found it relevant to combine factors to describe combined etiologies in a few cases.

We realize that such underlying pathology will not be definable in a proportion of cases, but we hope that the systematic approach will encourage searching the chart for the original pathology. The advantage would be to reduce the proportion of unhelpful “idiopathic” or “unknown,” which could benefit the individual patient’s treatment and clinical and translational research and improve the quality of epidemiological data. We find it noteworthy that we arrived at only 10% of our test cohort coded as unknown by a comprehensive search in patient records.

### Factor “C”—complications

Complications are intimately related to the history of previous interventions. A multitude of publications attests to the type and occurrence of surgical complications associated with the treatment of hydrocephalus. Our options for complications are in accordance with this vast, published experience. We have chosen to subdivide into complications (1) related to the functionality of diversion procedure (mechanical shunt failure, ICP-malregulation with functional shunt, functional ETV failure); (2) shunt surgery related complications, although these may secondarily result in mechanical shunt dysfunction, (infection, bleeding, skin defects, shunt displacement, CSF leakage); and (3) patient-related complications unrelated to the functionality of the CSF diversion procedure (pain, allergies). For mechanical shunt failure a further subdivision can be added with the description of the location of shunt failure (*p* = proximal, *v* = valve, *d* = distal).

Similar to the considerations described under “P,” a patient’s history can consist of an accumulation of one or more complications over time. Therefore, adding a numerator would also give a more comprehensive description.

1. C1(4) is the patient with 4 shunt revisions due to mechanical failure
2. C3 is the patient with a failed ETV procedure but no other complications
3. C1(7)C3(1)C4a(1) is the patient with seven shunt revisions due to mechanical failure, one failed ETV procedure and one shunt infection

### Factor “T”—time

Hydrocephalus can occur at any age and, in most cases, results in a subsequent chronic condition. Onset and current presentation may thus be separated by many years. Physiology differs vastly in infants compared to older children and adults. Etiologies differ across infants, children, adults, and the elderly. Clinical presentations vary with age. In order to encompass the duality of age of onset and current age, we defined the “*T*” factor as a combination of age and time of onset in five categories: fetal/congenital hydrocephalus (1), pediatric-onset hydrocephalus (2), suspected congenital/pediatric-onset hydrocephalus (3), adult-onset hydrocephalus (4), senior onset hydrocephalus (5). The following examples illustrate the system.

- 6(0) is a 6-year-old child with infantile hydrocephalus
- 35(1) is a 35-year-old patient with pediatric-onset hydrocephalus (also referred to as transitional hydrocephalus [38].
- 43(2) is a 43-year-old patient with probable infantile/pediatric-onset/transitional hydrocephalus. Patients with longstanding overt ventriculomegaly in adults (LOVA) belong to this group.
- 52(3) is a 52-year-old patient with adult-onset hydrocephalus. Many of these patients will have a clinical presentation of secondary NPH.
- 69(4) is a 69-year-old patient with “senior onset.” Patients with NPH will make up the majority of this group.

### Numeric coding vs. systematic narrative

Although intended to be helpfully unambiguous, a combination of numbers and letters is not intuitively understandable. In order to improve clinical usefulness, we thus suggest that a systematic narrative can supplement clinical communication [32]. Table 1 provides a “translation” between the numeric codes and a systematic narrative for each of the six factors. We propose that the numeric combinations could be more useful for administrative, epidemiological, and research purposes.

#### Preliminary results

A full set of coding for all six factors was possible for patients in the test cohort with both the numeric coding and the standardized narrative, which can be illustrated by the following two vignettes. The vignettes also show the difference between coding with the options of the ICD-10 and ICD-11 systems and the ASPECT options.

### Case 1

A 72-year-old man with no medical history presented with unsteady gait and several falls. The symptoms began 2 years prior and had worsened progressively. The patient described a sensation of his feet being difficult to lift, particularly when walking on stairs. Furthermore, the patient described short-term memory loss and decreased attention span.

Objectively, the patient had normal alertness, attention, and orientation. There was reduced strength in the lower extremities, positive catch, hyperreflexia, and clonus bilaterally. The gait was unstable, broad, and shuffling. MR cerebrum showed severe ventriculomegaly without clear obstructions.

#### ICD-10 classification

The following three ICD-10 codes can be applied: G91.0 (communicating hydrocephalus), G91.1 (obstructive hydrocephalus), G91.8 (other hydrocephalus), and G91.2 (normal pressure hydrocephalus).

#### ICD-11 classification

The following four ICD-11 diagnoses can be applied: 8D.64.04 (normal-pressure-hydrocephalus), 8D.64.0Y (other specified communicating hydrocephalus) and 8D64.1Y (other specified non-communicating hydrocephalus).

#### ASPECT Hydrocephalus system coding

The following coding is the only applicable. ASPECT: V2dS2A0, S3, P0, E9, C0, T4 (72).

### Case 2

A 17-year-old man with a known pineal tumor treated with a VP-shunt was admitted with headache for 1 week. During the last 24 h the patient had started vomiting. He had normal alertness, attention, and orientation at admission. CT showed dilated ventricular system including both lateral ventricles, third and fourth ventricle. The subarachnoid space was unaffected, and CT showed no additional abnormalities.

The patient was primarily treated with shunt implementation at age 12 and had since then undergone two shunt revisions and one EVD. The shunt revisions were due to a valve occlusion and skin defect.

The patient underwent another shunt revision due to a misplaced internal ventricular catheter.

#### ICD-10 classification

The following five ICD-10 codes can be applied: G91.1 (obstructive hydrocephalus), G91.8 (other hydrocephalus), G91.9 (hydrocephalus, unspecified), G94.1 (hydrocephalus in neoplastic disease and G94.1 (hydrocephalus in other diseases classified elsewhere).

#### ICD-11 classification

The following five ICD-11 diagnoses can be applied: 8D64.Z (hydrocephalus, unspecified), 8D64.1Z (non-communicating hydrocephalus, unspecified), 8D64.1Y (other specified non-communicating hydrocephalus), LA04.0 (hydrocephalus with stenosis of the aqueduct of Sylvius), and 8D64.10 (hydrocephalus due to structural malformations).

#### ASPECT Hydrocephalus System coding

The following coding is the only applicable. ASPECT: V2dS0A0, S1, P1(3)P3(1), E4, C1(2)C3c(1), T2(17).

The system will be further tested and validated on a larger consecutive cohort independent of the test cohort.

## Discussion

Multiple factors characterize hydrocephalus [4, 18, 21, 25, 28, 29, 35, 36]. A balance between classification accuracy and simplicity is hard to achieve in a system based on one or a few factors.

Our proposal for a new hydrocephalus descriptive system emerges from significant logical and functional limitations classifying hydrocephalus by ICD-10 and ICD-11 and from other published classification systems limiting the description to a single or two factors. The ASPECT Hydrocephalus System uses six factors in a parallel, unprioritized principle.

The structure of the ASPECT Hydrocephalus System provides several clinically useful things: (1) it ensures standardized coding of critical factors in hydrocephalus; (2) there is no forced hierarchy of factors allowing some factors to be individually more important; (3) the parallelism of coded factors means that missing one factor does not impede usefulness; (4) it serves as a checklist for the clinician to ensure that the most relevant factors are identified and considered. In addition, it may help educate patients on their disease and thus improve patient autonomy and further function as a “hydrocephalus passport,” smoothening patients’ transition between units and hospitals [2, 10, 38].

One intended advantage of a non-hierarchical system is that the importance and clinical relevance of factors may differ between patients and also over time for the same patient. The coding options are deliberately basic in order to make the system useful to perform a systematic and comprehensive patient description at all times and regardless of hydrocephalus expertise or access to advanced diagnostics. Some of the information may be unobtainable at the first patient encounter. As the ASPECT code does not require immediate completion, incomplete information does not thwart the system’s functionality. Intervention may, in some cases, be initiated before completion of all ASPECT factors, and the ASPECT code can be completed post-intervention. The ASPECT code contains dynamic data and should be re-evaluated when the patient is admitted or when a treatment decision is to be made. Thus, a patient may receive updated alternating ASPECT codes throughout life.

## Limitations

ASPECT Hydrocephalus System aims at global applicability providing a systematic and reproducible overview of any patient’s hydrocephalus history. This approach removes a need to use advanced or developing diagnostic tools not available everywhere spanning measurements of ICP, real-time imaging, biomarkers, and genetics [3, 12, 13, 18, 35, 36]. Additionally, the procedural invasiveness to, e.g., ICP monitoring and CSF sampling, is usually unnecessary for primary clinical assessment, but has proven usefulness for secondary, more advanced diagnostics [3]. Non-invasive methods of ICP measurement are emerging and becoming increasingly reliable but have yet to replace invasive ICP monitoring [14, 15, 20]. As the availability of these diagnostic methods increases and the clinical utility of the factors they examine have been validated, these factors can and should be added to the ASPECT Hydrocephalus System.

The ASPECT Hydrocephalus System does not function as a classification system, as classification is meant to group patients into categories. Such grouping may build on a single factor or a hierarchy of factors. These principles are deliberately avoided in the ASPECT Hydrocephalus System to encompass the clinical diversity of factors. It may, however, be possible to extract singular factors from ASPECT coded patient cohorts for classification purposes, e.g., by dichotomizing a patient cohort according to one factor, e.g., one of the complication types if one was to conduct a study on this complication type and outcome; or by extracting patients with transitional hydrocephalus looking for possible differences in educational and professional status compared to a non-hydrocephalic population. The “age and time-of-onset” factor might even become a future candidate as the classification principle in ICD and other systems with health administration purposes.

The ASPECT Hydrocephalus System is not a grading system for risk or severity in contrast to the numerical scores of, e.g., the Early Warning Score, CHA_2_DS_2_-VASc, and Glasgow Coma Scale [19, 31, 33]. Unfortunately, the multifactorial nature of hydrocephalus is incompatible with a meaningful single score grading. Thus, the ASPECT Hydrocephalus System is not useful for directly summarizing the total risk or severity of the disease, and each factor must be considered individually. The system does not rank factors and allows for ethical, personal, economic, and scientific factors to influence hydrocephalus management for the individual patient.

## Future perspectives

In order to ensure clinical reliability, this description of hydrocephalus and previous classification systems should be retrospectively and prospectively validated for intra- and interclinician reproducibility. Validating the system should include predefined quality measures, e.g., maximum allowed percentage of codings allocated to “unspecific” or “unknown” and minimum acceptable percentage of unapplicable coding. The validation should include centers that have not participated in designing the system. We expect some of the factors or the definition of these to be modified by the experience obtained in a validation process, and we further expect the system to undergo modifications by clinical use.

## Supplementary Information

Below is the link to the electronic supplementary material.

Supplementary file1 (DOCX 19 KB)
