# Correction to: The ASPECT Hydrocephalus System: a non‑hierarchical descriptive system for clinical use

**DOI:** 10.1007/s00701-022-05478-2

**Published:** 2023-01-10

**Authors:** Joachim Birch Milan, Thorbjørn Søren Rønn Jensen, Nicolas Nørager, Sarah Skovlunde Hornshøj Pedersen, Casper Schwartz Riedel, Nikolaj Malthe Toft, Ahmed Ammar, Mansoor Foroughi, André Grotenhuis, Andrea Perera, Harold Rekate, Marianne Juhler

**Affiliations:** 1 Copenhagen CSF Study Group, Copenhagen, Denmark; 2 Department of Neurosurgery 6031, Rigshospitalet, Inge Lehmanns Vej 6, 2100 Copenhagen, DK Denmark; 3 Department of Neurosurgery, King Fahd University Hospital, Imam Abdulrahman Bin Faisal University, Dammam, Saudi Arabia; 4 European Association of Neurosurgical Societies (EANS), CSF Task Force, Brussels, Belgium; 5 Department of Neurosurgery, Wellington Hospital, London, UK; 6 Department of Neurosurgery, Radboud University Nijmegen Medical Centre, Holland Nijmegen, Netherlands; 7 Department of Basic and Clinical Neuroscience, Kings College London, Maurice Wohl Clinical Neuroscience Institute, London, UK; 8 Department of Neurosurgery, Hofstra Northwell School of Medicine in Hempstead, Hempstead, NY USA; 9 Department of Clinical Medicine, University of Copenhagen, Copenhagen, Denmark

**Correction to: Acta Neurochirurgica**

**https://doi.org/10.1007/s00701-022-05412-6**

The correct author names should be: Casper Schwartz Riedel and Mansoor Foroughi.

The original article has been corrected.

