# Supplementary material for: The ASPECT Hydrocephalus System: a non-hierarchical descriptive system for clinical use
Source: Acta Neurochir (Wien). 2022 Nov 24;165(2):355–65. doi: 10.1007/s00701-022-05412-6 (PMC9922243; doi:10.1007/s00701-022-05412-6)
Supplement: Supplementary file 1 — Supplementary file1 (DOCX 19 KB) [file 701_2022_5412_MOESM1_ESM.docx]

## Methodology

Primarily, a research group specialized in hydrocephalus was formed. It consisted of young researchers, PhD students, post docs, one associate professor, and 2 professors, with different professional backgrounds relevant for the project (medical student, medical doctor, nurse, molecular biologist, biomedical engineer). In plenum, relevant factors for hydrocephalus were discussed. The research group agreed on eight relevant patient characteristics: ‘current age’, ‘obstruction/ventricular system morphology (radiologically suspected/confirmed)’, ‘time of onset’, ‘etiology (e.g., previous cerebral event)’, ‘symptoms’, ‘previous treatment (shunt, ETV, both, none)’, ‘ICP and/or ICP dynamics’, and ‘genetics and syndrome association’.

Second, a rating-system with the factors was developed and forwarded to an external panel of hydrocephalus expert, along with information explaining the rating-system. An expert in hydrocephalus was defined as a neurosurgeon with hydrocephalus as his/hers primary clinical or research area. The experts were asked to rank four priorities in treatment choice/conducting research. The named experts were:

- Ahmed Ammar, Department of Neurosurgery, King Fahd University Hospital, Faculty of Medicine, Imam Abdulrahman Bin Faisal University, Al Khobar, Saudi Arabia
- Uwe Kehler, Department of Neurosurgery, Unfallkrankenhaus Berlin, Berlin, Germany
- Harold Rekate, Hofstra Northwell School of Medicine in Hempstead, New York, USA
- Mark Hamilton, Department of Clinical Neurosciences, University of Calgary, Calgary, Alberta, Canada
- Mansoor Foroughi, Department of Neurosurgery, Wellington Hospital, London, United Kingdom

Every expert listed ‘obstruction/ventricular system morphology’ and ‘etiology’ amongst their top four priorities. However, there was no further consensus in prioritizing the characteristics. One listed ‘ICP and/or ICP dynamics’, one listed ‘time of onset’, two listed ‘symptoms’ and one listed ‘obstruction/ventricular system morphology’ as the most important patient characteristic in treatment choice (see table below for further details). Based on the heterogeny expert-rating, the research group agreed on a non-hierarchical system, in which all characteristics contribute equally to the description of the patient.

Third, the primary aim with the ASPECT system was to create a system that could be used world-wide, regardless of technically facilities available. Thus, it was decided that ‘ICP and/or ICP dynamics’ and ‘genetics and syndrome association’ should not be included in the system but could be add-on’s in hospitals with the proper facilities.

## Frequency tables

| Priority number | Chosen characteristics in treatment choice and their frequency |
| --- | --- |
| Priority 1 | Obstruction/ventricular system morphology (1/5)  Time of onset (1/5)  Symptoms (2/5)  ICP and/or ICP dynamics (1/5) |
| Priority 2 | Obstruction/ventricular system morphology (4/5)  Etiology (1/5) |
| Priority 3 | Etiology (3/5)  Previous treatment (2/5) |
| Priority 4 | Etiology (1/5)  Previous treatment (1/5)  ICP and/or ICP dynamics (2/5)  Genetics and syndrome association (1/5) |

| Priority number | Chosen characteristics in conducting research and their frequency |
| --- | --- |
| Priority 1 | Obstruction/ventricular system morphology (1/5)  Etiology (2/5)  ICP and/or ICP dynamics (1/5)  Genetics and syndrome association (1/5) |
| Priority 2 | Obstruction/ventricular system morphology (2/5)  Time of onset (1/5)  Etiology (1/5)  ICP and/or ICP dynamics (1/5) |
| Priority 3 | Obstruction/ventricular system morphology (1/5)  Time of onset (1/5)  Symptoms (1/5)  ICP and/or ICP dynamics (1/5)  Genetics and syndrome association (1/5) |
| Priority 4 | Current age (1/5)  Obstruction/ventricular system morphology (1/5)  Etiology (2/5)  ICP and/or ICP dynamics (1/5) |

Description of the iterative process to improve coherence and precision of the ASPECT Hydrocephalus system.

The theoretical ASPECT system was discussed several times in the research group and the EANS taskforce group, and subsequently revised.

At last, three authors (MJ, TJ, NMT) tested the ASPECT system on 50 randomly chosen patients. For every patient each factor was applied followed by a discussion and consensus in plenum. The revised ASPECT system was applied on a new set of patients and its functionality was subsequently discussed. The revision/discussion process was repeated until all factors of the ASPECT system could easily, and without exceptions, be applied to all patients in the test group.

Examples:

Factor “A” (Anatomy): the first version described dilated, normal and small/overdrained ventricles in an assumed symmetrical appearance. In the test group, there were two patients with unilateral overdrainage and dilatation of the opposite lateral ventricle, which led to addition of these options.

Factor “P” (Previous Interventions): the first version listed the type of interventions. The test cohort contained both patients with many interventions and patients with only one/a few previous interventions. This led to the addition of a numerator to highlight the complexity of the patient’s previous history.
